# MRC5 cells engineered to express ACE2 serve as a model system for the discovery of antivirals targeting SARS-CoV-2

**DOI:** 10.1038/s41598-021-84882-7

**Published:** 2021-03-08

**Authors:** Kentaro Uemura, Michihito Sasaki, Takao Sanaki, Shinsuke Toba, Yoshimasa Takahashi, Yasuko Orba, William W. Hall, Katsumi Maenaka, Hirofumi Sawa, Akihiko Sato

**Affiliations:** 1 Drug Discovery and Disease Research Laboratory, Shionogi & Co., Ltd, Osaka, Japan; 2 Division of Molecular Pathobiology, Research Center for Zoonosis Control, Hokkaido University, Sapporo, Japan; 3 Laboratory of Biomolecular Science, Faculty of Pharmaceutical Sciences, Hokkaido University, Sapporo, Japan; 4 Department of Immunology, National Institute of Infectious Diseases, Tokyo, Japan; 5 International Collaboration Unit, Research Center for Zoonosis Control, Hokkaido University, Sapporo, Japan; 6 National Virus Reference Laboratory, School of Medicine, University College of Dublin, Dublin, Ireland; 7 Global Virus Network, Baltimore, MD USA; 8 Center for Research and Education On Drug Discovery, Faculty of Pharmaceutical Sciences, Hokkaido University, Sapporo, Japan; 9 Global Station for Biosurfaces and Drug Discovery, Hokkaido University, Sapporo, Japan

**Keywords:** Microbiology, Virology, Antivirals, SARS-CoV-2

## Abstract

Although the spread of Severe Acute Respiratory Syndrome Coronavirus-2 (SARS-CoV-2) has resulted in a worldwide pandemic, there are currently no virus-specific drugs that are fully effective against SARS-CoV-2. Only a limited number of human-derived cells are capable of supporting SARS-CoV-2 replication and the infectivity of SARS-CoV-2 in these cells remains poor. In contrast, monkey-derived Vero cells are highly susceptibility to infection with SARS-CoV-2, although they are not suitable for the study of antiviral effects by small molecules due to their limited capacity to metabolize drugs compared to human-derived cells. In this study, our goal was to generate a virus-susceptible human cell line that would be useful for the identification and testing of candidate drugs. Towards this end, we stably transfected human lung-derived MRC5 cells with a lentiviral vector encoding angiotensin-converting enzyme 2 (ACE2), the cellular receptor for SARS-CoV-2. Our results revealed that SARS-CoV-2 replicates efficiently in MRC5/ACE2 cells. Furthermore, viral RNA replication and progeny virus production were significantly reduced in response to administration of the replication inhibitor, remdesivir, in MRC5/ACE2 cells compared with Vero cells. We conclude that the MRC5/ACE2 cells will be important in developing specific anti-viral therapeutics and will assist in vaccine development to combat SARS-CoV-2 infections.

## Introduction

Severe Acute Respiratory Syndrome Coronavirus-2 (SARS-CoV-2) emerged suddenly in December 2019 and rapidly spread to become a worldwide pandemic. SARS-CoV-2 is a member of the *Betacoronavirus* genus of the family *Coronaviridae*; it is closely related to the SARS-CoV that circulated worldwide in 2002 and 2003^1–3^. To date, several candidate compounds, including remdesivir, have been evaluated in clinical trials for the treatment of SARS-CoV-2-infected patients^4,5^. However, there are no currently-approved specific drugs directed against the SARS-CoV-2. As such, effective therapeutic agents as well as vaccines against the virus are urgently needed.

Cell-based assays are typically employed in the first steps in drug discovery. There are only a few human-derived cell lines, including lung-derived Calu-3, colon-derived Caco-2, and liver-derived Huh7 cells that are susceptible to infection with SARS-CoV-2; however, the infectivity of SARS-CoV-2 in each of these cell lines is ~ tenfold lower than that observed using Vero cells^6^. However, although monkey kidney-derived Vero cells are highly susceptible to infection with both SARS-CoV and SARS-CoV-2^6,7^, they exhibit comparatively weak antiviral responses to certain compounds, including remdesivir due to their low capacity for drug activation and metabolism compared with their human-derived counterparts^8,9^. To help resolve this, our goal was to engineer a human cell line that would be susceptible to SARS-CoV-2 infection and that it could be used to facilitate discovery of antiviral agents and vaccines against this virus.

Human angiotensin-converting enzyme 2 (hACE2) acts as an entry receptor for both SARS-CoV and SARS-CoV-2^10–12^; cell lines that are engineered to express hACE2 have been shown to be susceptible to infection with SARS-CoV. For example, human 293T and HeLa cells that express recombinant hACE2 have been used successfully in pseudovirus entry assays, in authentic virus infection assays, and for screening antiviral compounds^7,13,14^. Results from several studies have revealed that expression of hACE2 facilitates entry of SARS-CoV-2 into otherwise refractory 293T and HeLa cells^15,16^. These engineered cell lines have been used in research studies focused on repurposing U.S. Food and Drug Administration-approved small molecules and for the evaluation of antiviral effects of entry inhibitors in tests employing SARS-CoV-2 pseudoviruses^9,16,17^.

Human lung-derived MRC5 cells are highly susceptible to the infection of various human coronaviruses, including HCoV-OC43, HCoV-229E and Middle East respiratory syndrome coronavirus (MERS-CoV)^18–20^. In this study, we generated MRC5 cells that stably-expressed hACE2 and examined their susceptibility to SARS-CoV-2 infection and their capacity to support virus replication. In addition, we have employed the MRC5/ACE2 cells to evaluate antiviral activities of a number of small molecules, including remdesivir.

## Results and discussion

### Expression of exogenous human ACE2 confers susceptibility to SARS-CoV-2 infection on refractory cell lines

**Figure 1:**
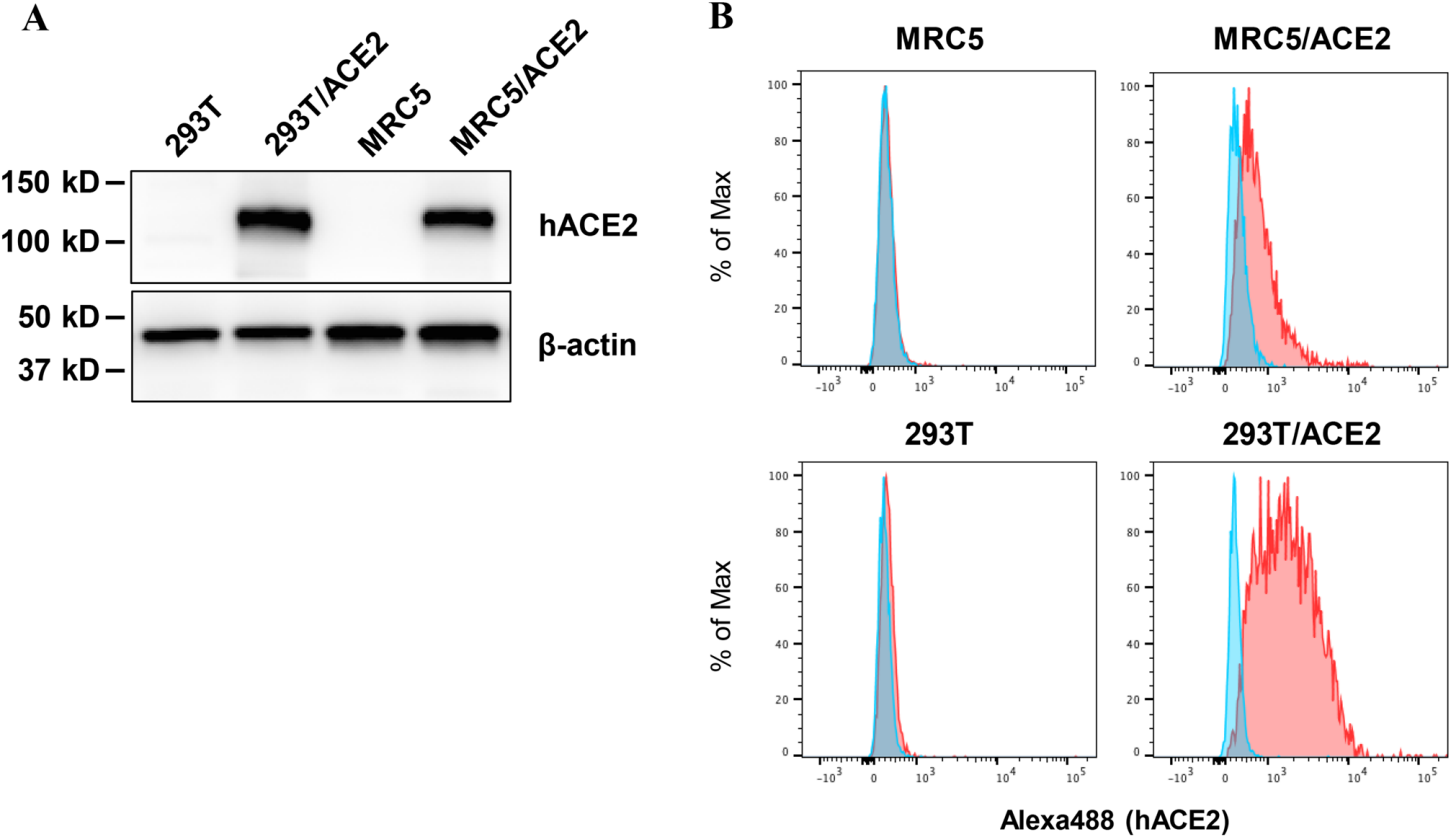
Expression of human ACE2. (**A**) Expression of immunoreactive human ACE2 (hACE2) in each of the lentiviral-transfected cell lines was examined using an anti-ACE2 antibody. Expression of β-actin was used as a loading control. Full-length blots are presented in Supplementary Figure S4. (**B**) Flow cytometric analysis of surface expression of hACE2 in MRC5, MRC5/ACE2, 293T and 293T/ACE2 cells using anti-ACE2 (red) or isotype control (blue) antibodies.

MRC5 cells are highly susceptible to infection with human coronaviruses 229E and OC43, but resistant to SARS-CoV and SARS-CoV-2^7,11,18,19^. Both MRC5 and 293T cells were transduced with a recombinant lentiviral vector to generate MRC5/ACE2 and 293T/ACE2 cells, respectively, that stably express recombinant hACE2. Expression of hACE2 was confirmed by immunoblotting, flow cytometry and immunofluorescence assays (IFAs) using an anti-human ACE2 antibody (Fig. 1A,B and Supplementary Figure S1A).

**Figure 2:**
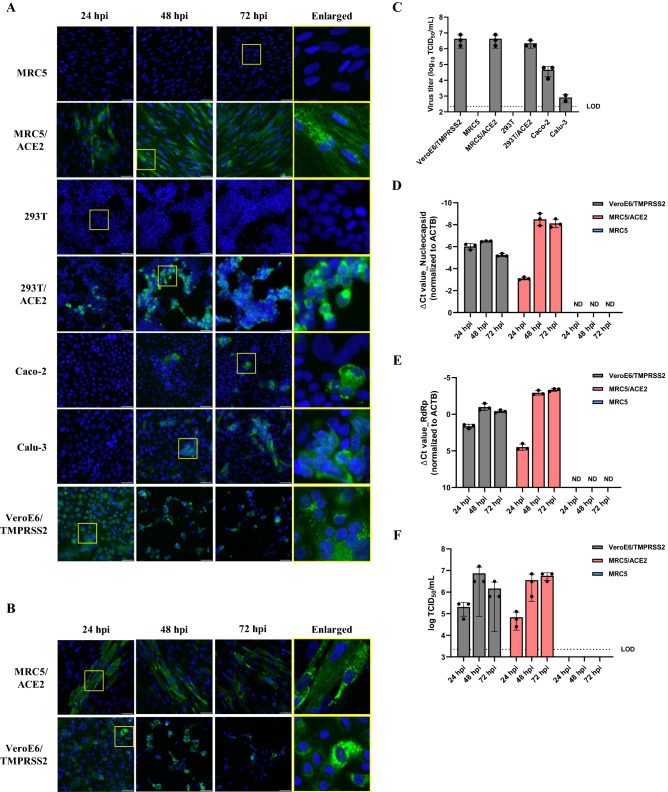
Replication and production of viral proteins in SARS-CoV-2-infected MRC5/ACE2 cells. (**A**) Cells were infected with SARS-CoV-2 at a multiplicity of infection (MOI) of 0.01 for 1 h. At 24, 48 and 72 hpi, cells were stained with anti-SARS-CoV-2 S (Spike protein) antibody (green) and counterstained with Hoechst 33342 nuclear dye (blue). Scale bars indicate 50 μm. (**B**) Cells were infected with SARS-CoV-2 at an MOI of 0.01 for 1 h. At 24, 48 and 72 hpi, cells were stained with anti-SARS-CoV-2 N (nucleocapsid protein) antibody (green) and counterstained with Hoechst 33342 nuclear dye (blue). Scale bars indicate 50 μm. (**C**) Cells were infected with SARS-CoV-2 at an MOI of 0.01 for 1 h. At 48 hpi, supernatants were collected; a monolayer of VeroE6/TMPRSS2 cells was inoculated with serial dilutions followed by incubation for 72 h. The infectious viral titers were measured via a calculation of the TCID_50_/ml. Data represent the average of three replicates from a single experiment, and error bars indicate standard deviation (SD). Dotted line indicates the limit of detection (LOD). (**D** and **E**) Cells were infected with SARS-CoV-2 at an MOI of 0.01 for 1 h. At 24, 48 and 72 hpi, the relative levels of sub-genomic (N) or viral genomic (RdRp) RNA were evaluated by qRT-PCR. The ACTB transcript was used as a reference control. Data represent the average of three replicates from a single experiment, and error bars indicating SD. ND indicate not detected. (**F**) Cells were infected with SARS-CoV-2 at an MOI of 0.01 for 1 h. At 24, 48 and 72 hpi, supernatants were collected; the infectious viral titers were measured by the same method as described above. Data represent the average of three replicates from a single experiment, and error bars indicate SD. Dotted line indicates the LOD.

We examined the susceptibility of MRC5/ACE2 and 293T/ACE2 cells to infection with SARS-CoV-2, and compared our results to those obtained when targeting Calu-3 and Caco-2 cells that express hACE2 constitutively, and with green monkey kidney-derived Vero E6 cells that stably express human type II transmembrane serine protease (VeroE6/TMPRSS2)^10,21–23^. At 24, 48 and 72 h post infection (hpi), SARS-CoV-2-infected cells were identified using an IFA with an anti-SARS-CoV-2 Spike (S) or nucleocapsid (N) protein antibody. Numerous SARS-CoV-2-S-positive MRC5/ACE2 and 293T/ACE2 cells were detected at 24 hpi, and time-dependent spread of infection was observed. In contrast, no infection was observed in cells of the parental MRC5 and 293T cells (Fig. 2A and Supplementary Figure S2A). Viral S protein antigen was widely detected in infected VeroE6/TMPRSS2 cells when compared with that of the Calu-3 and Caco-2 cells (Fig. 2A and Supplementary Figure S2A). These results are consistent with those in previous reports that revealed that VeroE6/TMPRSS2 cells are highly susceptible to SARS-CoV-2 infection^24^. Viral N protein was also widely detected in infected MRC5/ACE2 and VeroE6/TMPRSS2 cells (Fig. 2B and Supplementary Figure S2B). SARS-CoV-2 positive signals detected in MRC5/ACE2 cells were comparable to those identified in the VeroE6/TMPRSS2 cells results confirming that exogenous expression of hACE2 rendered the MRC5/ACE2 cells highly susceptible to SARS-CoV-2 infection. Earlier studies reported that no SARS-CoV-2 or SARS-CoV pseudovirus entry and no evidence for SARS-CoV infection could be observed in naïve MRC5 cells^7,11^. These findings suggested that resistance to SARS-CoV-2 and SARS-CoV infection in this cell line was directly associated with the absence of the ACE2 viral entry receptor.

### Amplification of SARS-CoV-2 in MRC5/ACE2 cells is more efficient than in virus-susceptible human cell lines

We then also examined the viral progeny yield in SARS-CoV-2-infected MRC5/ACE2 and 293T/ACE2 cells. We also examined these parameters in VeroE6/TMPRSS2, Calu-3, and Caco-2 cells that have previously been confirmed to be susceptible to SARS-CoV-2 infection^6,24^. Culture supernatants of SARS-CoV-2-infected cells were collected at 48 hpi and viral titers in the supernatants were evaluated by cytopathic effect (CPE) and quantified via calculation of tissue culture infectious dose (TCID)_50_/ml. Higher virus titers from culture supernatants of infected MRC5/ACE2 and 293T/ACE2 cells were detected; no virus titers were detected in the supernatants of SARS-CoV-2-challenged MRC5 and 293T cells (Fig. 2C). Consistent with the results obtained with IFA, higher virus titers were detected in supernatants from SARS-CoV-2-infected VeroE6/TMPRSS2 cells compared with those from the infected Calu-3 and Caco-2 cells (Fig. 2C). Virus titers in the supernatants from SARS-CoV-2-infected MRC5/ACE2 cells were comparable to those from infected VeroE6/TMPRSS2 cells and were higher than those detected from Calu-3 and Caco-2 cells. These findings are consistent with previous reports, suggesting that these cells are all susceptible to SARS-CoV-2 infection^6,24^.

We then quantified viral RNA replication and viral progeny yield in SARS-CoV-2-infected MRC5, MRC5/ACE2 and VeroE6/TMPRSS2 cells at multiple time points. At 24, 48 and 72 hpi, both the SARS-CoV-2 nucleocapsid gene (a marker for sub-genomic RNA replication) and the RNA-dependent RNA polymerase gene (RdRp; a marker for genomic RNA replication) were quantified by qRT-PCR analysis. qRT-PCR analysis revealed that viral RNA was increased in SARS-CoV-2-infected MRC5/ACE2 cells. In contrast, no infection was observed in cells of the parental MRC5 (Fig. 2D,E). Progeny virus titers from culture supernatants of infected MRC5/ACE2 cells correlated well with the results of qRT-PCR; no virus titers were detected in the supernatants of SARS-CoV-2-challenged MRC5 cells (Fig. 2F). Higher levels of viral RNA replication, including both the nucleocapsid and RdRp genes, and virus titers were detected in VeroE6/TMPRSS2 cells at 24 hpi compared to those in MRC5/ACE2 cells (Fig. 2D–F). After 48 hpi, a strong virus-induced CPE was observed in the SARS-CoV-2-infected VeroE6/TMPRSS2 cells, but subsequently viral RNA number and progeny virus titers were decreased after 48 hpi. Taken together, these results demonstrated that expression of exogenous hACE2 rendered MRC5 cells highly susceptible to SARS-CoV-2 infection.

While SARS-CoV-2 can replicate in Calu-3 and Caco-2 cells, the number of viral antigen-positive cells and titers of virus progeny were much lower compared to infected MRC5/ACE2 cells (Fig. 2). As such, although primary infection rates in Calu-3 and Caco-2 cells were low, these cells did facilitate efficient replication of SARS-CoV-2 (Figs. 2C and Supplementary Figures S2C–F). Furthermore, although human-derived Calu-3 and Caco-2 cells have been identified as susceptible to SARS-CoV-2 infection, these cells display markedly reduced sensitivity compared to both Vero E6 and VeroE6/TMPRSS2 cells^6,24^. When engaged in targeted drug development for human diseases, it is important to conduct cell-based assays using human-derived cells or tissues as the capacity for specific drug metabolism may be somewhat species-specific. This was shown clearly in experiments in which the replication inhibitor, remdesivir, was evaluated in monkey kidney Vero E6 cells^8,9^. Taken together, our results suggested that MRC5/ACE2 cells may facilitate a comprehensive evaluation of virus replication and of potential drugs which might be used to target SARS-CoV-2 infection.

### SARS-CoV-2-infected MRC5/ACE2 cells exhibit sensitivity to antiviral compounds

**Figure 3:**
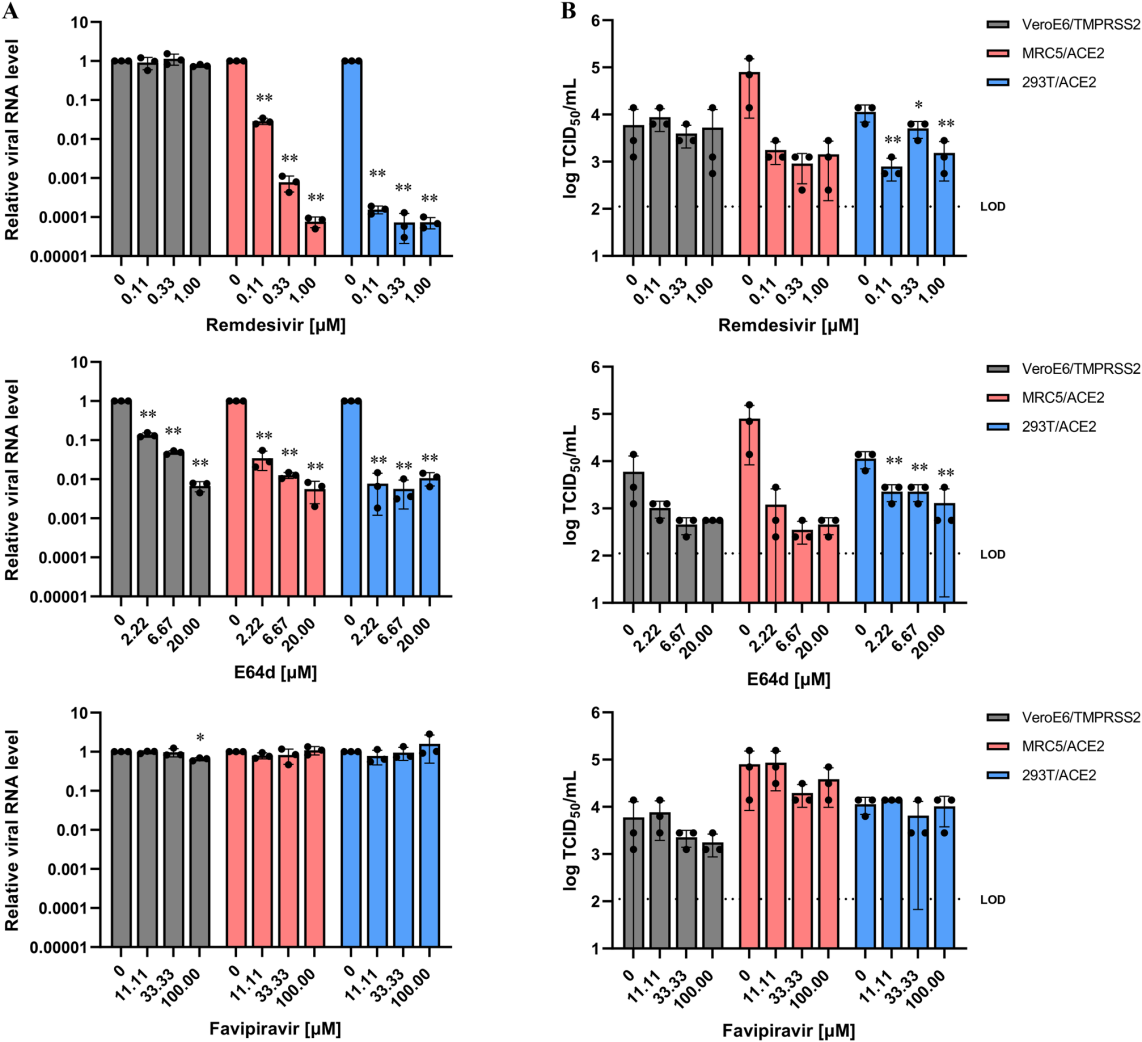
SARS-CoV-2 infection of MRC5/ACE2 cells and sensitivity to antiviral agents. (**A**) VeroE6/TMPRSS2, MRC5/ACE2 and 293T/ACE2 cells were all treated with remdesivir (0.11, 0.33 and 1 μM, red bars), E64d (2.22, 6.67 and 20 μM, blue bars) or favipiravir (11.11, 33.33 and 100 μM, green bars) for 30 min prior to infection with SARS-CoV-2 at an MOI of 0.1. At 24 hpi, relative expression of the nucleocapsid gene was evaluated by qRT-PCR with β-actin mRNA used as a reference control. Data represent the average of three replicates from a single experiment, and error bars indicate SD. Statistically significant differences were determined with a one-way ANOVA followed by Dunnett’s multiple comparisons test; *P < 0.05 and **P < 0.0001. (**B**) Supernatants from cultures described in (**A**) were collected at 24 hpi; serial dilutions were prepared and used to inoculate a monolayer of VeroE6/TMPRSS2 cells. The infectious viral titers were measured at 72 hpi via calculation of the TCID_50_/ml. Data represent the average of three replicates from a single experiment, and error bars indicate SD. Dotted line indicates the LOD. Statistically significant differences were determined with a one-way ANOVA followed by Dunnett’s multiple comparisons test; *P < 0.05 and **P < 0.01.

Antiviral activity depends in large part on the capacity for drug uptake and activation / metabolism and this can vary widely, depending on the target cells used in these assays. As such, we evaluated the efficacy of several compounds with documented anti-SARS-CoV-2 activity in antiviral assays in MRC5/ACE2 cells. Remdesivir and favipiravir are both nucleoside analogue prodrugs that inhibit viral RNA synthesis via a delayed chain termination mechanism^25–27^. Likewise, E64d is a cathepsin B/L inhibitor that prevents viral entry via the inhibition of endosome-virus membrane fusion^11,28^. VeroE6/TMPRSS2, MRC5/ACE2 and 293T/ACE2 cells were infected with SARS-CoV-2 in the presence of remdesivir (0.11, 0.33 and 1 μM), favipiravir (11.11, 33.33 and 100 μM) or E64d (2.22, 6.67 and 20 μM) and intracellular levels of viral RNA and production of infectious viral particles were examined at 24 hpi. Analysis by qRT-PCR revealed dose-dependent reductions in viral RNA synthesis in MRC5/ACE2 and 293T/ACE2 cells in response to remdesivir. However, VeroE6/TMPRSS2 cells were relatively insensitive to the antiviral effects of remdesivir (Fig. 3A; upper graph). In contrast, addition of favipiravir resulted in a suppression of viral RNA replication in SARS-CoV-2-infected VeroE6/TMPRSS2 cells at 100 μM but both MRC5/ACE2 and 293T/ACE2 cells were relatively insensitive to the antiviral effects of favipiravir (Fig. 3A; bottom graph). Addition of E64d resulted in a significant decrease in viral RNA in all cells evaluated (Fig. 3A; middle graph). Likewise, and similar to the findings from the qRT-PCR analysis, our results revealed more than 1-log reduction in progeny virus titers in supernatants of SARS-CoV-2-infected MRC5/ACE2 cells treated with remdesivir. In contrast, the impact of remdesivir was limited in virus-infected VeroE6/TMPRSS2 cells (Fig. 3B).

Remdesivir-mediated antiviral activity against coronaviruses varies and is directly dependent on the cell line targeted. For example, a recent report revealed that the anti-SARS-CoV-2 activity of remdesivir was six-fold higher in infected Calu-3 cells (EC_50_ of 0.28 μM) compared to Vero E6 cells (EC_50_ of 1.65 μM)^8^; another report revealed that the anti-SARS-CoV-2 activity of remdesivir was 86-fold higher in infected 293T/ACE2 cells (EC_50_ of 0.0072 μM) than in Vero E6 cells (EC_50_ of 0.62 μM)^9^. Similarly, remdesivir-mediated antiviral activity against human coronavirus 229E strain was 196-fold higher in Huh7 cells (EC_50_ of 0.02 μM) than that in porcine kidney-derived LLC-PK1 cells (EC_50_ of 3.8 μM)^29^. A similar phenomenon was reported in experiments employing sofosbuvir, a nucleotide prodrug, with activity against Zika virus (ZIKV)-infected Vero cells. In this report, sofosbuvir-mediated anti-ZIKV activity in Huh7 cells exhibited an EC_50_ of 4 μM; however the EC_50_ in Vero cells was substantially greater (i.e., > 50 μM)^30^.

Remdesivir and sofosbuvir are both phosphoramidate prodrugs; they require activation via sequential hydrolysis catalyzed by intracellular esterases, including carboxylesterase 1, cathepsin A and histidine triad nucleotide-binding protein 1; the actions of these enzymes convert the pharmacologically inactive drug into the activated nucleoside triphosphate form that promotes the antiviral effect^31^. Earlier reports suggested that some cell lines, including Vero and LLC-PK1, may be deficient with regard to the cellular metabolic machinery required to activate nucleoside phosphoramidate prodrugs. As such, remdesivir is considered to be a nucleotide analog prodrug that is activated in esterase-rich human-derived cell lines or tissues.

At this time, most experiments focused on the screening of candidate anti-SARS-CoV-2 compounds employ Vero or Vero-related cell lines^25,32–34^. As some highly effective agents require metabolic activation and thus can exert an effective antiviral effect in human cell lines only, there is an urgent need to develop strong and well-characterized human cell line-based assay systems that can be used to complement the “gold-standard” assays using Vero cells. The use of multiple cell lines will serve to increase assay sensitivity and facilitate effective and efficient screening. As shown, human lung-derived MRC5 cells are highly susceptible to the infection of various human coronaviruses, including HCoV-OC43, HCoV-229E and MERS-CoV, and can be used to facilitate discovery of anti-coronavirus drugs^19,20,29,35,36^. In the studies carried out here, we revealed that MRC5/ACE2 cells are highly susceptible to SARS-CoV-2 infection and support robust replication. We have also shown that this cell line is suitable for the study of antiviral effects by small molecules. As such, the employment of MRC5/ACE2 cells may make an important contribution in the development of both broad-spectrum as well as SARS-CoV-2-specific antiviral drugs and/or vaccines.

## Methods

### Cells

MRC5 cells (American Type Culture Collection, Manassas, VA, USA) and Caco-2 cells (RIKEN BRC, Ibaraki, Japan) were maintained in Minimum Essential Medium GlutaMAX Supplement (Gibco; Thermo Fisher Scientific, Waltham, MA, USA) supplemented with 10% fetal bovine serum (FBS, Gibco), nonessential amino acids (Wako, Osaka, Japan), sodium pyruvate (Wako), and penicillin–streptomycin (P/S, Wako) at 37 °C. Vero E6 (ATCC), 293T (RIKEN BRC) and Calu-3 (ATCC) cells were maintained in high-glucose Dulbecco’s modified Eagle’s medium (Gibco) supplemented with 10% FBS and P/S at 37℃.

### Generation of TMPRSS2- and ACE2-expressing cells

Vero E6 cells stably expressing human TMPRSS2 (VeroE6/TMPRSS2) were generated by lentiviral transduction with CSII-CMV-TMPRSS2-IRES2-Bsd and blasticidin-based selection. MRC5 and 293T cells stably expressing human ACE2 (MRC5/ACE2 and 293T/ACE2) were generated by lentiviral transduction with pLVSIN-CMV-ACE2-Pur and puromycin-based selection. For lentiviral vector preparation, 293T cells were co-transfected with the aforementioned lentiviral vector plasmid and Lentiviral High Titer Packaging Mix (Takara Bio, Shiga, Japan).

### Virus

SARS-CoV-2 strain JPN/TY/WK-521, a clinical isolate from a COVID-19 patient^24^ was kindly provided by Dr. Masayuki Shimojima (National Institute of Infectious Diseases, Tokyo, Japan). The virus was amplified in VeroE6/TMPRSS2 cells with Mynox mycoplasma elimination reagent (Minerva Biolabs, Berlin, Germany). The viral titers were measured by inoculating VeroE6/TMPRSS2 cells with five-fold serial dilutions of virus; CPE was scored in order to calculate the TCID_50_/ml.

### Antiviral compounds

GS-5734 (Remdesivir) was purchased from MedChemExpress (Monmouth Junction, NJ, USA). E64d was supplied by FUJIFILM Wako Pure chemical (Osaka, Japan). Favipiravir was supplied by PharmaBlock Sciences, Inc. (Nanjing, China). All compounds were solubilized in 100% dimethyl sulfoxide (DMSO, Sigma-Aldrich, St. Louis, MO, USA) for in vitro studies.

### Western blotting

Cells were lysed in Sample Buffer Solution (Nacalai tesque, Kyoto, Japan), heated at 95℃ for 10 min, and subjected to sodium dodecyl sulfate – polyacrylamide gel electrophoresis (SDS-PAGE). Proteins were transferred from the gels onto Immobilon-P PVDF membranes (Millipore, Burlington, MA, USA). ACE2 and β-actin protein were detected on blots by probing with anti-hACE2 antibody (#4355, Cell Signaling Technologies, Danvers, MA, USA) and anti-actin antibody (MAB1501, Chemicon, Temecula, CA, USA), respectively. Immune complexes were detected using horseradish peroxidase (HRP)-conjugated secondary antibodies and SuperSignal West Femto Maximum Sensitivity Substrate (Pierce; Thermo Fisher Scientific).

### Flow cytometry

Cells were harvested with Cell Dissociation Buffer (Gibco) and incubated with anti-hACE-2 antibody (AF933, R&D Systems, Minneapolis, MN, USA) or goat-derived anti-mouse IgG (A11032, Invitrogen; Thermo Fisher Scientific) isotype control. Cells expressing hACE2 were detected using Alexa Fluor 488-conjugated anti-goat IgG antibody on a FACS Canto flow cytometer (BD Biosciences, San Jose, CA, USA). Data were analyzed with FlowJo version 10.6.0 (BD Biosciences).

### Real-time quantitative reverse transcription PCR (qRT-PCR)

SARS-CoV-2 was handled in Biosafety level 3 (BSL3) facilities throughout. Each cell line was seeded into wells in 48-well plates on the day prior to infection; cells were then infected with SARS-CoV-2 at an MOI of 0.01 for 1 h. After incubation, unbound viruses were removed, and fresh medium was added. At 24, 48 and 72 hpi, total RNA was isolated using a PureLink RNA Mini Kit (Ambion; Thermo Fisher Scientific) and quantified by real-time qRT-PCR analysis using an EXPRESS One-step SuperScript qRT-PCR kit (Invitrogen) and a QuantStudio 7 Flex Real-Time PCR system (Applied Biosystems; Thermo Fisher Scientific). The primers and probe sequences (Integrated DNA Technologies, Coralville, IA, USA) targeting the SARS-CoV-2 nucleocapsid gene to detect sub-genomic viral RNA and the RdRp gene to detect viral genomic RNA were described in previous report^37^. The primers and probe for ACTB (Hs01060665_g1, Applied Biosystems) transcripts were used as internal controls.

### Virus replication assay

Each cell line was seeded into wells of 48-well plates on the day prior to virus infection; cells were then infected with SARS-CoV-2 at an MOI of 0.01. The virus inoculum was removed after 1 h of incubation; cells were washed twice with culture medium and fresh medium was added. At 24, 48 and 72 hpi, supernatants were collected and serial dilutions were prepared; dilutions were used to inoculate a monolayer of VeroE6/TMPRSS2 cells. At 72 hpi, viral titers were determined by calculation of the TCID_50_/ml.

### Immunofluorescence assay

Each cell line seeded into wells in 48-well plates on the day prior to virus infection; cells were then infected with SARS-CoV-2 at an MOI of 0.01 or 0.1 for 1 h. At 24, 48 and 72 hpi, cells were fixed with Masked Form A (Japan Tanner Co., Osaka, Japan), permeabilized with ice-cold methanol and stained with anti-SARS-CoV-2 Spike antibody (1A9; GTX632604, GeneTex, Irvine, CA, USA), SKOT-8 antibody (anti-SARS-CoV N)^38^, anti-hACE2 antibody (R&D Systems), Alexa Fluor Plus 488-conjugated anti-mouse IgG antibody and 594-conjugated anti-goat IgG (Invitrogen). Cell nuclei were counterstained with Hoechst 33342 (Molecular Probes, Eugene, OR, USA). Cells were then evaluated by fluorescence microscopy (IX73, Olympus, Tokyo, Japan). Images were processed with cellSens Standard 1.16 (Olympus).

### Sensitivity to antiviral agents

VeroE6/TMPRSS2, MRC5/ACE2 and 293T/ACE2 cells were seeded into wells of 96-well plates. Each were treated with remdesivir (0.11, 0.33 and 1 μM), E64d (2.22, 6.67 and 20 μM) or favipiravir (11.11, 33.33 and 100 μM) for 30 min prior to infection with SARS-CoV-2. Cells were then infected with SARS-CoV-2 at an MOI of 0.1. At 24 hpi, total RNA was isolated using a PureLink 96 total RNA Purification Kit (Invitrogen), and viral RNA was quantified by qRT-PCR analysis as described above. Supernatants were collected, and serial dilutions were used to inoculate a monolayer of VeroE6/TMPRSS2 cells. Three days after inoculation, CPE was scored, and TCID_50_/ml was calculated to measure viral titers.

### Statistical analysis

One-way ANOVA followed by Dunnett’s multiple comparisons test was performed to determine statistical significance using GraphPad Prism version 8.4.2 (GraphPad Software, La Jolla, CA, USA).

## Supplementary Information

Supplementary Information
