# Supplementary material for: MRC5 cells engineered to express ACE2 serve as a model system for the discovery of antivirals targeting SARS-CoV-2
Source: Sci Rep. 2021 Mar 8;11:5376. doi: 10.1038/s41598-021-84882-7 (PMC7940632; doi:10.1038/s41598-021-84882-7)
Supplement: Supplementary file 1 — Supplementary Information [file 41598_2021_84882_MOESM1_ESM.docx]

**MRC5 cells engineered to express ACE2 serve as a model system for the discovery of antivirals targeting SARS-CoV-2**

Kentaro Uemura^1,2,3^, Michihito Sasaki^2^, Takao Sanaki^1,2^, Shinsuke Toba^1,2^, Yoshimasa Takahashi^4^, Yasuko Orba^2,5^, William W. Hall^5,6,7^, Katsumi Maenaka^3,8,9^, Hirofumi Sawa^2,5,7^, Akihiko Sato^1,2 *^

1 Drug Discovery and Disease Research Laboratory, Shionogi & Co., Ltd., Osaka, Japan

2 Division of Molecular Pathobiology, Research Center for Zoonosis Control, Hokkaido University, Sapporo, Japan

3 Laboratory of Biomolecular Science, Faculty of Pharmaceutical Sciences, Hokkaido University, Sapporo, Japan

4 Department of Immunology, National Institute of Infectious Diseases, Tokyo, Japan

5 International Collaboration Unit, Research Center for Zoonosis Control, Hokkaido University, Sapporo, Japan

6 National Virus Reference Laboratory, School of Medicine, University College of Dublin, Ireland

7 Global Virus Network, Baltimore, Maryland, USA

8 Center for Research and Education on Drug Discovery, Faculty of Pharmaceutical Sciences, Hokkaido University, Sapporo, Japan

9 Global Station for Biosurfaces and Drug Discovery, Hokkaido University, Sapporo, Japan

*Corresponding author:

Akihiko Sato

E-mail: [akihiko.sato@shionogi.co.jp](mailto:akihiko.sato@shionogi.co.jp)

**Supplementary Figure S1**

**
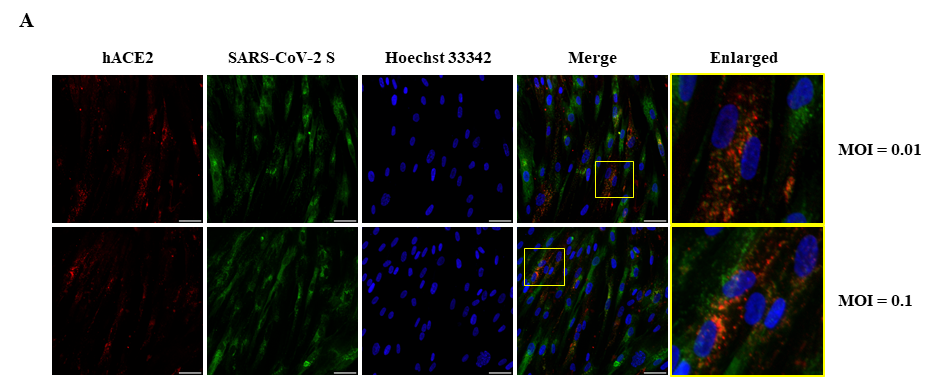
**

**Supplementary Figure S1. Expression of human ACE2 and SARS-CoV-2 S protein**

**(A)** MRC5/ACE2 cells were infected with SARS-CoV-2 at an MOI of 0.1 or 0.01. At 48 hpi, cells were stained with anti-ACE2 antibody (red), anti-SARS-CoV-2 S (Spike protein) antibody (green) and Hoechst 33342 nuclear dye (blue). Scale bars indicate 50 μm.

**Supplementary Figure S2**

**
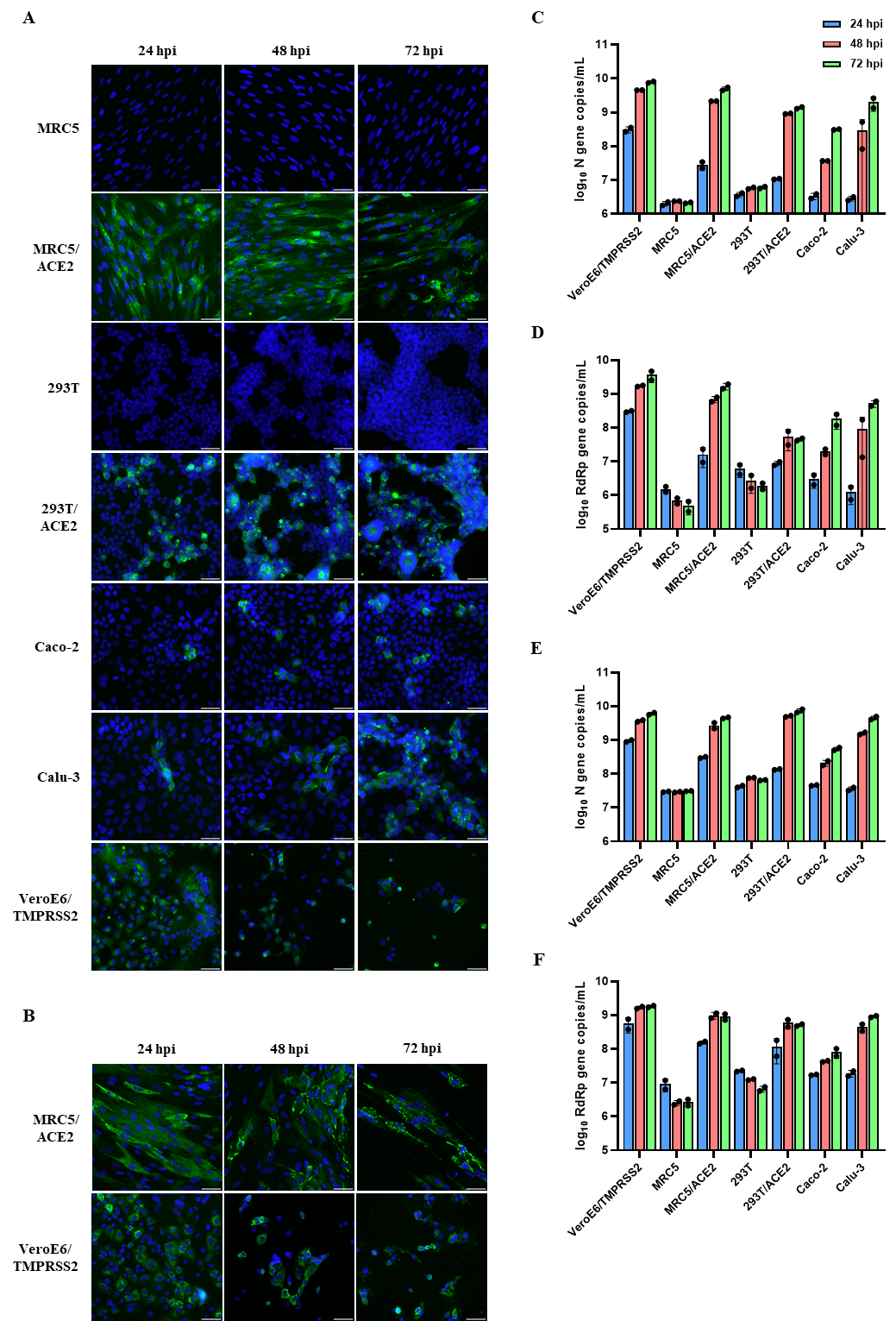
**

**Supplementary Figure S2. Replication and production of viral proteins in SARS-CoV-2-infected cells**

**(A)** Cells were infected with SARS-CoV-2 at a multiplicity of infection (MOI) of 0.1 for 1 h. At 24, 48 and 72 hpi, cells were stained with anti-SARS-CoV-2 S (Spike protein) antibody (green) and counterstained with Hoechst 33342 nuclear dye (blue). Scale bars indicate 50 μm.

**(B)** Cells were infected with SARS-CoV-2 at an MOI of 0.1 for 1 h. At 24, 48 and 72 hpi, cells were stained with anti-SARS-CoV-2 N (nucleocapside protein) antibody (green) and counterstained with Hoechst 33342 nuclear dye (blue). Scale bars indicate 50 μm.

**(C and D)** Cells were infected with SARS-CoV-2 at an MOI of 0.01 for 1 h. At 24, 48 and 72 hpi, culture supernatants were collected; viral sub-genomic (N, Figure S2C) or genomic (RdRp, Figure S2D) RNA copies were evaluated by qRT-PCR. Data represent the average of two replicates from a single experiment, and error bars indicating SD.

**(E and F)** Cells were infected with SARS-CoV-2 at an MOI of 0.1 for 1 h. At 24, 48 and 72 hpi, supernatants were collected; viral sub-genomic (N, Figure S2E) or genomic (RdRp, Figure S2F) RNA copies were evaluated by qRT-PCR. Data represent the average of two replicates from a single experiment, and error bars indicating standard deviation SD.

**Supplementary Figure S3**


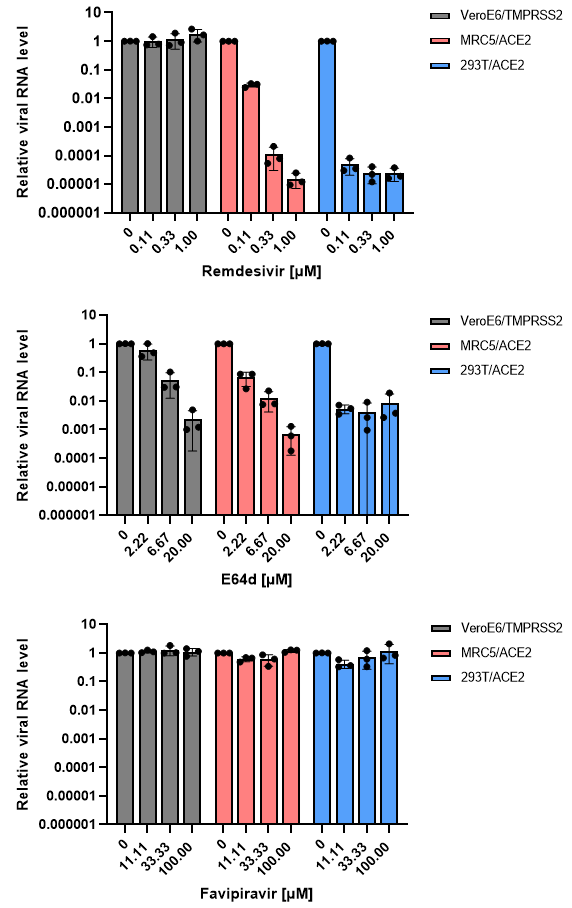


**Supplementary Figure S3. SARS-CoV-2 infection of MRC5/ACE2 cells and sensitivity to antiviral agents**

VeroE6/TMPRSS2, MRC5/ACE2 and 293T/ACE2 cells were all treated with remdesivir (0.11, 0.33 and 1 μM), E64d (2.22, 6.67 and 20 μM) or favipiravir (11.11, 33.33 and 100 μM) for 30 min prior to infection with SARS-CoV-2 at an MOI of 0.1. At 48 hpi, relative expression of the nucleocapside gene was evaluated by qRT-PCR with β-actin mRNA used as a reference control. Data represent the average of three replicates from a single experiment, and error bars indicate SD.

**Supplementary Figure S4**


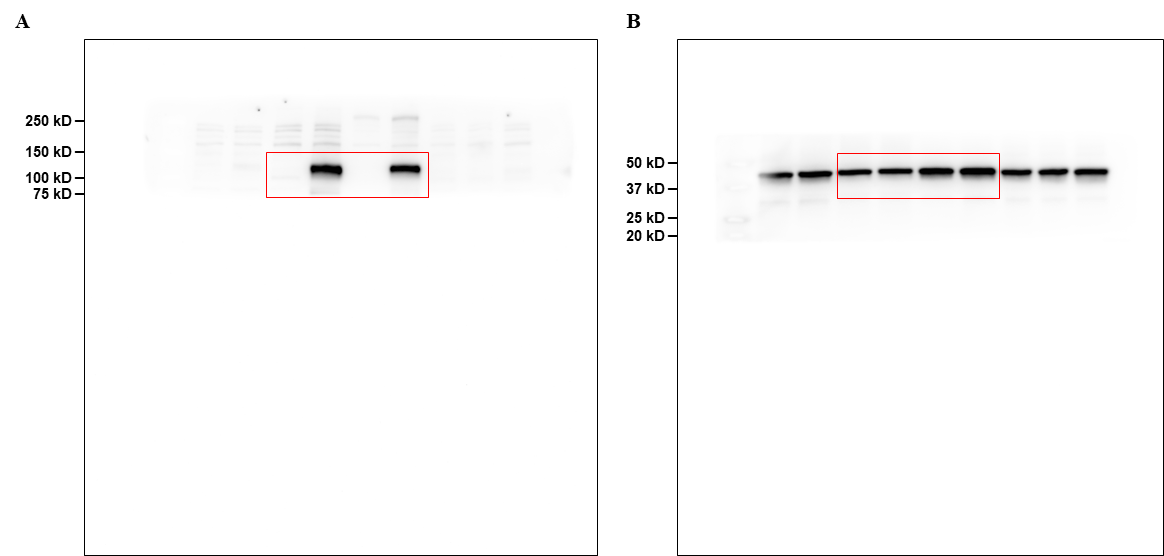


**Supplementary Figure S4. Full-length image for Figure 1A in the manuscript text**

Uncropped image of the blots against human ACE2 (A) and β-actin (B). The boxed regions in Figures S4A and S4B are presented in the indicated figures in the manuscript text.
